# Auditory and Somatosensory Interaction in Speech Perception in Children and Adults

**DOI:** 10.3389/fnhum.2019.00344

**Published:** 2019-10-04

**Authors:** Paméla Trudeau-Fisette, Takayuki Ito, Lucie Ménard

**Affiliations:** ^1^Laboratoire de Phonétique, Université du Québec à Montréal, Montreal, QC, Canada; ^2^Centre for Research on Brain, Language and Music, Montreal, QC, Canada; ^3^GIPSA-Lab, CNRS, Grenoble INP, Université Grenoble Alpes, Grenoble, France; ^4^Haskins Laboratories, Yale University, New Haven, CT, United States

**Keywords:** multisensory integration, speech perception, auditory and somatosensory feedback, adults, children, categorization, maturation

## Abstract

Multisensory integration (MSI) allows us to link sensory cues from multiple sources and plays a crucial role in speech development. However, it is not clear whether humans have an innate ability or whether repeated sensory input while the brain is maturing leads to efficient integration of sensory information in speech. We investigated the integration of auditory and somatosensory information in speech processing in a bimodal perceptual task in 15 young adults (age 19–30) and 14 children (age 5–6). The participants were asked to identify if the perceived target was the sound /e/ or /ø/. Half of the stimuli were presented under a unimodal condition with only auditory input. The other stimuli were presented under a bimodal condition with both auditory input and somatosensory input consisting of facial skin stretches provided by a robotic device, which mimics the articulation of the vowel /e/. The results indicate that the effect of somatosensory information on sound categorization was larger in adults than in children. This suggests that integration of auditory and somatosensory information evolves throughout the course of development.

## Introduction

From our first day of life, we are confronted with multiple sensory inputs such as tastes, smells, and touches. Unconsciously, related inputs are combined into a single input with rich information. Multisensory integration (MSI), also called multimodal integration, is the ability of the brain to assimilate cues from multiple sensory modalities that allows us to benefit from the information from each sense to reduce perceptual ambiguity and ultimately reinforce our perception of the world (Stein and Meredith, 1993; Stein et al., 1996; Robert-Ribes et al., 1998; Molholm et al., 2002). MSI holds a prominent place in the way that information is processed, by shaping how inputs are perceived. This merging of various sensory inputs into common neurons was typically assumed to occur late in the perceptual process stream (Massaro, 1999), but recent studies in neurophysiology have even demonstrated that MSI can occur in the early stages of cortical processing, even in brain regions typically associated with lower-level processing of uni-sensory inputs (Macaluso et al., 2000; Foxe et al., 2002; Molholm et al., 2002; Mishra et al., 2007; Raij et al., 2010; Mercier et al., 2013).

While some researchers have suggested that an infant’s brain is likely equipped with multisensorial functionality at birth (Bower et al., 1970; Streri and Gentaz, 2004), others have suggested that MSI likely develops over time as a result of experiences (Birch and Lefford, 1963; Yu et al., 2010; Burr and Gori, 2011). Several studies support the latter hypothesis. For example, studies have demonstrated that distinct sensory systems develop at different rates and in different ways, which suggests that several mechanisms are implicated in MSI depending on the type of interactions (Walker-Andrews, 1994; Gori et al., 2008; Burr and Gori, 2011; Dionne-Dostie et al., 2015). For example, researchers have reported that eye-hand coordination, a form of somatovisual interaction, can be observed in infants as young as a week old (Bower et al., 1970), and audiovisual association of phonetic information emerges around 2 months of age (Kuhl and Meltzoff, 1982; Patterson and Werker, 2003), but audiovisual integration in spatial localization behavior does not appear before 8 months of age (Neil et al., 2006).

Ultimately, although it is still unclear whether an innate system enables MSI in humans, data from infants, children, and adults suggest that unimodal and multimodal sensory experiences and brain maturation enables the establishment of efficient integration processing (Rentschler et al., 2004; Krakauer et al., 2006; Neil et al., 2006; Gori et al., 2008; Nardini et al., 2008; Hillock et al., 2011; Stein et al., 2014) and that multisensory tasks in school-aged and younger children are executed through unimodal dominance rather than integration abilities (McGurk and Power, 1980; Hatwell, 1987; Misceo et al., 1999; Burr and Gori, 2011). Moreover, according to the intersensory redundancy hypothesis, perception of multimodal information is only facilitated when information from various sources is redundant, and not when the information is conflicting (Bahrick and Lickliter, 2000, 2012).

Multimodal integration is crucial for speech development. According to the associative view, during infancy, the acoustic features of produced and perceived speech are associated with felt and seen articulatory movements required for their production (Kuhl and Meltzoff, 1982; Patterson and Werker, 2003; Pons et al., 2009; Yeung and Werker, 2013). Once acoustic information and proprioceptive feedback information are strongly linked together, this becomes part of an internal multimodal speech model (Guenther and Perkell, 2004; Tourville and Guenther, 2011; Guenther and Vladusich, 2012).

MSI can sometimes be overlooked in speech perception since speakers frequently have one dominant sensory modality (Hecht and Reiner, 2009; Lametti et al., 2012). However, even though audition is the dominant type of sensory information in speech perception, many researchers have suggested that other sensory modalities also play a role in speech processing (Perrier, 1995; Tremblay et al., 2003; Skipper et al., 2007; Ito et al., 2009; Lametti et al., 2012). The McGurk effect, a classic perceptual illusion resulting from incongruent simultaneous auditory and visual cues about consonants clearly demonstrates that information from multiple sensory channels is unconsciously integrated during speech processing (McGurk and MacDonald, 1976).

In the current study, we examined the integration of auditory and somatosensory interaction in speech perception. Previous research has suggested that to better understand how different types of sensory feedback interact in speech perception, we need to better understand how and when this becomes mature.

Hearing is one of the first sensory modalities to emerge in humans. While still *in utero*, babies can differentiate speech from non-speech and distinguish variability in speech length and intensity (for a review on auditory perception in the fetus, see Lecanuet et al., 1995). After birth, babies are very soon responsive to various rhythmic and intonation sounds (Demany et al., 1977) and can distinguish phonemic features such as voicing, manner, and place of articulation (Eimas et al., 1971). Specific perceptual aspects of one’s first language, such as sensitivity to phonemes and phonotactic properties, are refined by the first year of life (Kuhl, 1991). Although auditory abilities become well established in the early years of life, anatomical changes and experiences will guide the development of auditory skills throughout childhood (Arabin, 2002; Turgeon, 2011).

Little is known about the development of oral somatosensory abilities in typically developing children. Yet, some authors have worked on the development of oral stereognosis in children and adults, where stereognosis is the ability to perceive and recognize the form of an object in the absence of visual and auditory information, by using tactile information. In oral stereognosis, the form of an object is recognized by exploring tactile information such as texture, size or spatial properties, in the oral cavity. This is usually evaluated by comparing the ability of children and adults to differentiate or identify small plastic objects in their mouths. Researchers have reported that oral sensory discrimination skills depend on age (McDonald and Aungst, 1967; Dette and Linke, 1982; Gisel and Schwob, 1988). McDonald and Aungst (1967) showed that 6- to 8-year-old children correctly matched half of the presented forms; 17- to 31-year-old adolescents and adults had perfect scores; and scores declined significantly with age among the 52- to 89-year-olds. Dette and Linke (1982) found similar results in 3- to 17-year-olds. The effect of age was also found in younger vs. older children. Kumin et al. (1984) showed that among 4- to 11-year-olds, the older children had significantly better oral stereognosis scores than younger children. Gisel and Schwob (1988) reported that 7- and 8-year-old children had better identification skills in an oral stereognosis experiment than 5- and 6-year-old children. Interestingly, only the 8-year-old children showed a learning effect, in that they got better scores as the experiment progressed.

To explain this age-related improvement in oral stereognosis, it was suggested that oral stereognosis maturity is achieved when the growth of the oral and facial structures is complete (McDonald and Aungst, 1967; Gisel and Schwob, 1988). This explanation is consistent with vocal tract growth data that shows that while major changes occur in the first 3 years of life (Vorperian et al., 1999), important growth of the pharyngeal region is observed between puberty and adulthood (Fitch and Giedd, 1999) and multidimensional maturity of the vocal tract is not reached until adulthood (Boë et al., 2007, 2008).

A few recent studies have suggested that there is a link between auditory and somatosensory information in multimodal integration.

Lametti et al. (2012) proposed that sensory preferences in the specification of speech motor goals could mediate responses to real-time manipulations, which would explain the important variability in compensatory behavior to an auditory manipulation (Purcell and Munhall, 2006; Villacorta et al., 2007; MacDonald et al., 2010). They point out that one’s own auditory feedback is not the only reliable source of speech monitoring and, in line with the internal speech model theory, that somatosensory feedback would also be considered in speech motor control. In agreement with this concept, Katseff et al. (2012) suggested that partial compensation in auditory manipulation of real-time speech could be because both auditory and somatosensory feedback system monitor speech motor control and therefore, the two systems are competing when large sensory manipulation affects only one of the sensory channels.

A recent study of speech auditory feedback perturbations in blind and sighted speakers supports the latter explanation. It showed that typically developing adults, whose somatosensory goals are narrowed by vision were more likely to tolerate large discrepancies between the expected and produced auditory outcome, whereas blind speakers, whose auditory goals had primacy over somatosensory ones, tolerated larger discrepancies between their expected and produced somatosensory feedback. In this sense, blind speakers were more inclined to adopt unusual articulatory positions to minimize divergences of their auditory goals (Trudeau-Fisette et al., 2017).

Researchers have also suggested that acoustic and somatosensory cues are integrated. As far as we know, Von Schiller (cited in Krueger, 1970; Jousmäki and Hari, 1998) was the first one to report that sound could modulate touch. Indeed, although he was mainly focused on the interaction between auditory and visual cues, he showed in his 1932s article that auditory stimuli, such as tones and noise bursts, could influence an object’s physical perception. Since then, studies have shown how manipulations of acoustic frequencies or even changes in their prevalence can influence the tactile perception of objects, events, and skin deformation such as their perceived smoothness, occurrence, or magnitude (Krueger, 1970; Jousmäki and Hari, 1998; Guest et al., 2002; Hötting and Röder, 2004; Ito and Ostry, 2010). Multimodal integration was stronger when both perceptual sources were presented simultaneously (Jousmäki and Hari, 1998; Guest et al., 2002).

This interaction between auditory and tactile channels is also found in the opposite direction, in that somatosensory inputs can influence the perception of sounds. For example, Schürmann et al. (2004) showed that vibrotactile cues can influence the perception of sound loudness. Later, Gick and Derrick (2009) demonstrated that aerotactile inputs could modulate the perception of a consonant’s oral property.

Somatosensory information coming from orofacial areas is somewhat different from those typically intended. Kinesthetic feedback usually refers to information retrieved from position, movement, and receptors in muscles and articulators (Proske and Gandevia, 2009). However, some of the orofacial regions involved in speech production movement are devoid of muscle proprioceptors. Therefore, the somatosensory information guiding our perception and production abilities likely also come from cutaneous mechanoreceptors (Johansson et al., 1988; Ito and Gomi, 2007; Ito and Ostry, 2010).

Although many studies have reported on the role of somatosensory information derived from orofacial movement in speech production (Tremblay et al., 2003; Nasir and Ostry, 2006; Ito and Ostry, 2010; Feng et al., 2011; Lametti et al., 2012), few studies have reported its role in speech perception.

Researchers recently investigated the contribution of somatosensory information on speech perception mechanisms. Ito et al. (2009) designed a bimodal perceptual task experiment where they asked participants to identify if the perceived target was the word “head” or “had.” When the acoustic targets (all members of the “head/had” continuum) were perceived simultaneously to a skin manipulation recalling the oral articulatory gestures implicated in the production of the vowel /ϵ/, the identification rate of the target “head” was significantly improved. The researchers also tested different directions of the orofacial muscle manipulation and established that the observed effect was only found if the physical manipulation reflected a movement required in speech production (Ito et al., 2009).

Somatosensory information appears to even be involved in the processing of higher-level perceptual concepts (Ogane et al., 2017). In a similar perceptual task, participants were asked to identify if the perceived acoustic target was “l’affiche” (the poster) or “la fiche” (the form). The authors showed that the appropriate temporal positions of somatosensory skin manipulation in the stimulus word, simulating somatosensory inputs concerning the hyperarticulation of either the vowel /a/ or the vowel /i/, could affect the categorization of the lexical target.

Although further study would reinforce these findings, these experiments highlight the fact that the perception of linguistic inputs can be influenced by the manipulation of cutaneous receptors involved in speech motion (Ito et al., 2009, 2014; Ito and Ostry, 2010), and furthermore, attest of a strong link between auditory and somatosensory channels within the multimodal aspect of speech perception in adults.

The fact that sounds discrimination if facilitated when included in the infants’ babbling register (Vihman, 1996) is surely part of the growing body of evidence that demonstrates how somatosensory information that is derived from speech movement also influences speech perception in young speakers (DePaolis et al., 2011; Bruderer et al., 2015; Werker, 2018). However, to our knowledge, only two studies have investigated how somatosensory feedback is involved in speech perception abilities in children (Yeung and Werker, 2013; Bruderer et al., 2015). In both studies, the researchers manipulated oral somatosensory feedback by constraining tongue or lip movement, thus forcing the adoption of a precise articulatory position. Although MSI continues to evolve until late childhood (Ross et al., 2011), these two experiments in toddlers shed light on how this phenomenon emerges.

In their 2013 article, Yeung and Werker (2013) reported that when 4- and 5-month-old infants were confronted with incongruent auditory and labial somatosensory cues, they were more likely to fix the visual demonstration corresponding to the vowel perceived through the auditory channel. In contrast, congruent auditory and somatosensory cues did not call for the need to add a corresponding visual representation of the perceived vowel.

Also using a looking-time procedure, Bruderer et al. (2015) focused on the role of language experience on the integration of somatosensory information. They found that the ability of 6-month-old infants to discriminate between the non-native dental /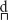/ and the retroflex /ɖ/ Hindi consonant was influenced by the insertion of a teething toy. When the toddlers’ tongue movements were restrained, they showed no evidence of phonetic contrast discrimination of tongue tip position. As shown by Ito et al. (2009), the effect of somatosensory cues was only observed if the perturbed articulator would have been involved in the production of the sound that was heard.

While these two studies mainly focused on perceptual discrimination rather than categorical representation of speech, they suggest that proprioceptive information resulting from static articulatory perturbation plays an important role in speech perception mechanisms in toddlers and that the phenomenon of multimodal integration in the perception-production speech model starts early in life. The authors suggested that, even at a very young age, babies can recognize that information can come from multiple sources and they react differently when the sensory sources are compatible. However, it is still unknown when children begin to integrate various sensory sources to treat them as a single sensory source.

In the current study, we aimed to investigate how dynamic somatosensory information from orofacial cutaneous receptors is integrated in speech processing in children compared to adults. Based on previous research, we hypothesized that: (1) when somatosensory inputs are presented simultaneously with auditory inputs, this affects their phonemic categorization; (2) auditory and somatosensory integration is stronger in adults than in children; and (3) MSI is facilitated when both types of sensory feedback are consistent.

## Materials and Methods

### Participants

We recruited 15 young adults (aged 19–30), including eight females. We also recruited 21 children (aged 4–6) and after excluding seven children due to equipment malfunction (1), non-completion (2), or inability to understand the task (4), this left 14 children (aged 5–6) including 10 females, for the data analysis. Five- to six-year-old is a particularly interesting age window since children master all phonemes of their native language. However, they have not yet entered the fluent reading stage, during which explicit teaching of reading has been shown to alter multimodal perceptual (Horlyck et al., 2012).

All participants were native speakers of Canadian French and were tested for pure-tone detection threshold using an adaptive method (DT < 25 dB HL at 250, 500, 1,000, 2,000, 4,000 and 8,000 Hz). None of the participants reported having speech or language impairments. The research protocol was approved by the Université du Québec à Montréal’s Institutional Review Board (no 2015-05-4.2) and all participants (or the children’s parents) gave written informed consent. The number of participants was limited due to the age of the children and the length of the task (3 different tasks were executed on the same day).

### Experimental Procedure

As in the task used by Ito et al. (2009), the participants were asked to identify the vowel they perceived and were asked to choose between /e/ and /ø/. Based on Ménard and Boe (2004), the auditory stimulus consisted of 10 members of a synthesized /e–ø/ continuum generated using the Maeda model (see Table 1). This continuum was created such that the first four formants were equally distributed from those corresponding to the natural endpoint tokens of /e/ and /ø/. To ensure that the children understood the difference between the two vocalic choices, the vowel /e/ was represented by an image of a fairy (/e/ as in fée) and the vowel /ø/ was represented by an image of a fire (/ø/ as in feu). Since, we wanted to minimize large head movements during the experiment, the children were asked to point out the image corresponding to their answers. Both images were placed in front of them at shoulder level, three feet away from each other on the horizontal plane. The adults were able to use the keyboard without looking at it and they used the right and left arrows to indicate their responses.

**Table 1:** Formant and bandwidth values of the synthesized stimuli used in the perceptual task.

	Formant values					Bandwidths values				
	F1	F2	F3	F4	F5	B1	B2	B3	B4	B5
Auditory stimuli										
1	364	1,922	2,509	3,550	4,000	48	55	60	50	100
2	364	1,892	2,469	3,500	4,000	48	55	60	50	100
3	364	1,862	2,429	3,450	4,000	48	55	60	50	100
4	364	1,832	2,389	3,400	4,000	48	55	60	50	100
5	364	1,802	2,349	3,350	4,000	48	55	60	50	100
6	364	1,772	2,309	3,300	4,000	48	55	60	50	100
7	364	1,742	2,269	3,250	4,000	48	55	60	50	100
8	364	1,712	2,229	3,200	4,000	48	55	60	50	100
9	364	1,682	2,189	3,150	4,000	48	55	60	50	100
10	364	1,652	2,149	3,100	4, 000	48	55	60	50	100

**Figure 1:**
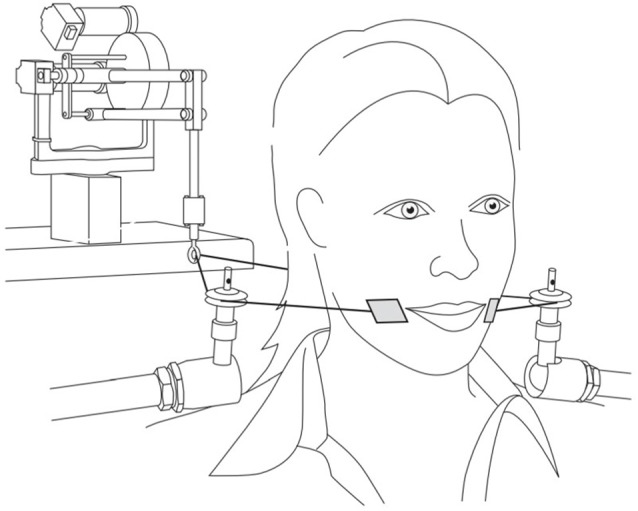
Experimental set up for facial skin stretch perturbations (reproduced with permission from Ito and Ostry, 2010).

Figure 1 shows the experimental set-up for the facial skin stretch perturbations. The participants were seated with their backs to a Phantom 1.0 device (SensAble Technologies) and they wore headphones (Sennheiser HD 380 pro). This small unit, composed of a robotic arm to which a wire is attached, allows for minor lateral skin manipulation at the side of the mouth, where small plastic tabs (2 mm × 3 mm), located on the ends of the wire, were placed with double-sided tape. The robotic arm was programed to ensure that when a four Newton flexion force was administered it led to a 10- to 15-mm lateral skin stretch.

When this facial skin stretch is applied at lateral to the oral angle in the backward direction as shown in the figure, it mimics the articulation associated with the production of the unrounded vowel /e/. Therefore, auditory and somatosensory feedback was either congruent (with /e/-like auditory inputs) or incongruent (with /ø/-like auditory inputs). As stated early, cutaneous receptors found in the within the labial area provides speech related kinesthetic information (Ito and Gomi, 2007). Since the skin manipulation was programed to be perceived at the same time as the auditory stimuli, it was possible to investigate the contribution of the somatosensory system to the perceptual processing of the speech targets.

The auditory stimuli were presented in 20 blocks of 10 trials each. Within each block, all members of the 10-step continuum were presented in a random order. For half of the trials, only the auditory stimulus was presented (unimodal condition). For the other half of the trials, a facial skin manipulation was also applied (bimodal condition). Alternate blocks of unimodal and bimodal conditions were presented to the participants. In total, 200 perceptual judgments were collected, 100 in the auditory-only condition and 100 in the combined auditory and skin-stretch condition.

### Data Analysis

For each participant, stimulus, and condition, we calculated the percentage of /e/ responses. The experiment was closely monitored, and the responses in trials where a short pause was requested by the participant were excluded from the analysis. In doing so, we sought to eliminate categorical judgments for which the participants were no longer in a position to properly respond to the task (fewer than 1.1% and 0.2% of all responses were excluded for children and adults, respectively). These perceptual scores were then fitted onto a logistic regression model (Probit model) to obtain psychometric functions from which the labeling slopes and 50% crossover boundaries were computed. The value of the slope corresponds to the sharpness of the categorization (the lower the value, the more distinct the categorization), while the boundary value indicates the location of the categorical boundary between the two vowel targets (the higher the value, the more toward /ø/ the frontier). Using the lme4 package in R, we carried out a linear mixed-effects model (Baayen et al., 2008) for both the steepness of the slopes and the category boundaries in which group (adult or children) and condition (unimodal or bimodal) were specified as fixed factors and individual participant was defined as a random factor.

Each given answer (5,800 perceptual judgments collected from 29 participants) was fitted into a linear mixed-effects model where fixed factors included stimuli (the 10-step continuum), group (adult or children), and condition (unimodal or bimodal), and the random factor was the individual participant. The mean categorization of the first and last two stimuli was also compared. Once again, the averages of the given answers (116 mean perceptual judgments collected from 29 participants) were fitted into a linear mixed-effects model where the fixed variables included stimuli (head stimuli or tail stimuli), group (adult or children), and condition (unimodal or bimodal) and where the random variable was the individual participant. Finally, independent *t*-tests were carried out in order to compare variability in responses between both experimental groups and conditions. In both cases, Kolmogorov–Smirnov tests indicated that categorizations followed a normal distribution.

## Results

**Figure 2:**
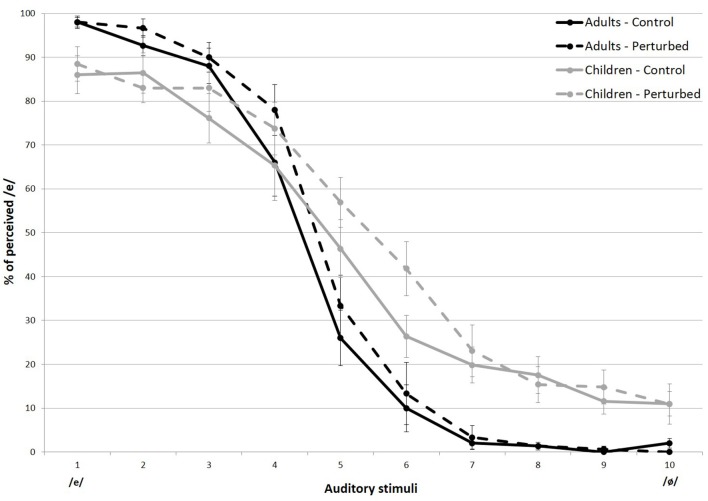
Percent identification of the vowel [e] for stimuli on the [e–ø] continuum, in both experimental conditions, for both groups. Error bars indicate standard errors.

**Figure 3:**
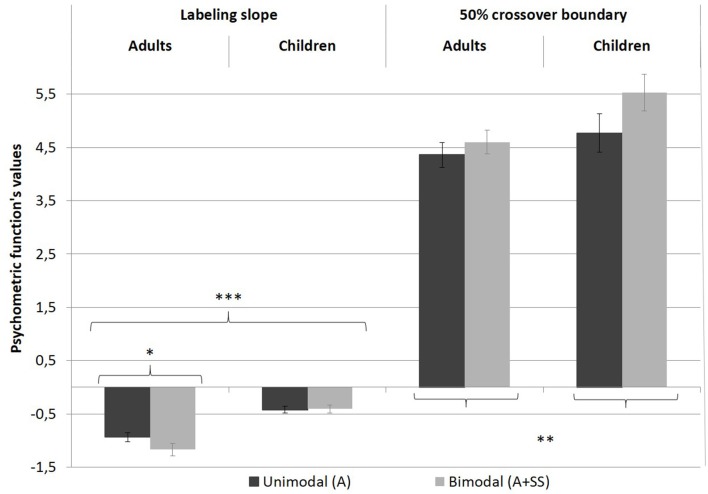
Psychometric functions of labeling slope and 50% crossover boundary, in both experimental conditions, for both groups. Error bars indicate standard errors. *p < 0.05; **p < 0.01; ***p < 0.001.

The overall percentage of /e/ responses for each stimulus is shown in Figure 2. The data were averaged across speakers, within both groups. Figure 3 displays the values for the labeling slope (distinctiveness of the vowels’ categorization) and 50% crossover boundary (location of the categorical frontier) averaged across experimental conditions and groups. As can be seen in both figures, regardless of the experimental condition, the children had greater variations in overall responses compared to the adults, which was confirmed in an independent *t*-test (*t*_(38)_ = 2.792, *p* < 0.01).

### Psychometric Functions

#### Labeling Slope Results

The linear mixed-effects model revealed a significant main effect of group on the steepness of the slope ($\chi_{\left(1\right)}^{2}$ = 23.549, *p* < 0.001), indicating that there was more categorical perception in adults than in children (see Figure 2, black lines and Figure 3, left-hand part of the graph).

Although no effect of condition as a main effect was observed ($\chi_{\left(1\right)}^{2}$ = 3.618, *p* > 0.05), a significant interaction between group and condition was found ($\chi_{\left(1\right)}^{2}$ = 4.956, *p* < 0.05). *Post hoc* analysis revealed that in the bimodal condition the slope of the labeling function was more abrupt for the adults (*z* = −3.153, *p* < 0.01) but not for the children, suggesting that the skin stretch condition led to a more categorical identification of the stimuli in adults only.

#### The 50% Crossover Boundary Results

A linear mixed-effects model analysis carried out on the 50% crossover boundaries revealed a single main effect of condition ($\chi_{\left(1\right)}^{2}$ = 9.245, *p* < 0.01). For both groups, the skin stretch perturbation led to a displacement of the 50% crossover boundary. In the bimodal condition (A+SS), the boundary was located closer to /ø/ than in the unimodal condition (A). This result is consistent with the expected effect of the skin stretch perturbation; more stimuli were perceived as /e/ than /ø/. No effect of group, as a main effect or with condition was found. The results are presented in Figure 2 and in Figure 3, in the right-hand part of the graphs.

### Categorical Judgments

A linear mixed-effects model analysis performed on the categorical judgments revealed that in addition to the expected main effect of stimuli ($\chi_{\left(1\right)}^{2}$ = 3652.4, *p* < 0.001), there were significant effects of group ($\chi_{\left(1\right)}^{2}$ = 4.586, *p* < 0.05) and condition ($\chi_{\left(1\right)}^{2}$ = 15.736, *p* < 0.001), suggesting that children and adults did not categorize the stimuli in a similar manner and that both experimental conditions prompted different categorization. Moreover, a significant interaction of group and stimuli ($\chi_{\left(1\right)}^{2}$ = 144.52, *p* < 0.001) revealed that irrespectively of the experimental condition, some auditory stimuli were categorized differently by the two groups.

*Post hoc* tests revealed that whether a skin stretch manipulation was applied or not, stimulus 7 (A *z* = −3.795, *p* < 0.1 A+SS *z* = −4.648, *p* < 0.01), 8 (A *z* = −3.445, *p* < 0.5 A+SS *z* = −3.544, *p* < 0.1) and 9 (A *z* = −3.179, *p* < 0.5 A+SS *z* = −4.347, *p* < 0.01) were more systematically identified as /ø/ by the adults than by the children. While no other two-way interactions were found, a significant three-way interaction of group, condition, and stimuli was observed ($\chi_{\left(4\right)}^{2}$ = 117.26, *p* < 0.001) suggesting that, for some specific stimuli, the skin stretch condition affected the perceptual judgment of both groups in a different manner.

First, it was found that the skin stretch manipulation had a greater effect on stimulus 6, in children only (*z* = −3.251, *p* < 0.5). For this group, the skin stretch condition caused a 15.8% increase of /e/ labeling on stimulus 6. For the adults, the addition of somatosensory cues only led to a 3.3% increase in /e/ categorization.

Although less expected, the skin stretch manipulation also led to some perceptual changes at the endpoint of the auditory continuum. As shown in Figure 2, stimulus 2 (*z* = 3.053, *p* < 0.5) and stimulus10 (*z* = −3.734, *p* < 0.1) were labeled differently by the two groups, but only in the bimodal condition. In fact, stimulus 2 (an /e/-like stimulus) was more likely to be identified as an /e/ by the adults in the experimental condition. In contrast, children were less inclined to label it so. As for stimulus 10 (an /ø/-like stimulus), the addition of somatosensory inputs decreased the correct identification rate in children only. In adults, although it barely affected their categorical judgments, the skin stretch manipulation mimicking the articulatory gestures of the vowel /e/ resulted in an increase of /ø/ labeling, as if it had a reverse effect.

Last, a comparison of mean categorizations of the first and last two stimuli revealed a main effect of stimuli ($\chi_{\left(1\right)}^{2}$ = 313.52, *p* < 0.001) and a significant interaction of group and stimuli ($\chi_{\left(1\right)}^{2}$ = 36.260, *p* < 0.001). More importantly, it also revealed a 3-way interaction of group, condition, and stimuli ($\chi_{\left(4\right)}^{2}$ = 37.474, *p* < 0.001). *Post hoc* tests indicated that those endpoint stimuli of the continuum were categorized differently by the two groups, but only when a skin stretch manipulation was applied. In agreement with previous results, in the skin stretch condition, children labeled more /e/-like stimuli as /ø/ (*z* = 3.434, *p* < 0.5), and more /ø/-like stimuli as /e/ (*z* = −4.139, *p* < 0.01).

## Discussion

This study aimed to investigate how auditory and somatosensory information is integrated in speech processing by school-aged children and adults, by testing three hypotheses.

As hypothesized, the overall perceptual categorization of the auditory stimuli was affected by the addition of somatosensory manipulations. The results for psychometric functions and categorical judgments revealed that auditory stimuli perceived simultaneously with skin stretch manipulations were labeled differently than when they were perceived on their own. Sounds were more perceived as /e/ when they were accompanied by the proprioceptive modification.

The second hypothesis that auditory and somatosensory integration would be greater in adults than in children was also confirmed. As shown in Figures 2, 3, orofacial manipulation affected the position of the 50% crossover boundary of both groups; when backward skin stretches were perceived simultaneously with the auditory stimulus, it increased its probability of being identified as an /e/. This impact of skin stretch manipulation on the value corresponding to the 50th percentile was also reported in Ito et al.’s (2009) experiment. However, bimodal presentation of auditory and somatosensory inputs affected the steepness of the slope in adults only. Figure 2 also shows that adult participants were more likely to label /e/-like stimuli as /e/ in the bimodal condition. Since negligible changes were observed for /ø/-like stimuli, it led to a more categorical boundary between the two acoustic vocalic targets. This difference in the integration patterns between children and adults suggests that linkage of specific somatosensory inputs with a corresponding speech sound evolves with age.

The third hypothesis that MSI would be stronger when auditory and somatosensory information was congruent was confirmed in adults but not in children. Only adults’ perception was facilitated when both sensory information was consistent. In children, a decrease in the correct identification rate resulted from the bimodal presentation when auditory and proprioceptive inputs were compatible. Moreover, while adults seemed to not be affected by the /e/-like skin stretches when auditory stimuli were alongside the prototypical /ø/ vocalic sound (see Figure 2), children’s categorization was influenced even when sensory channels were clearly contrasting, as if the bimodal presentation of vocalic targets blurred the children categorization abilities. Moreover, thought somatosensory information mostly affected specific stimuli in adult, it’s effect in children was further distributed along the auditory continuum. These last observations support our second hypothesis that MSI is strongly defined in adults.

As many have suggested, MSI continues to develop during childhood (e.g., Ross et al., 2011; Dionne-Dostie et al., 2015). The fact that young children are influenced by somatosensory inputs in a different manner then adults could, therefore, be due to their underdeveloped MSI abilities. Related findings have been reported for audiovisual integration (McGurk and MacDonald, 1976; Massaro, 1984; Desjardins et al., 1997). It has also been demonstrated that the influence of visual articulators in audition is weaker in school-aged children than in adults.

In agreement with the concept that MSI continues to develop during childhood, the differences observed between the two groups of perceivers could also be explained by the fact that different sensory systems develop at different rates and in different ways. In that sense, it has also been found that school-aged children were not only less likely to perceive a perceptual illusion resulting from incongruent auditory and visual inputs, but they also had poorer results in the identification of unimodal visual targets (Massaro, 1984).

Studies of the development of somatosensory abilities also support this concept. As established earlier, oral sensory acuity continues to mature until adolescence (McDonald and Aungst, 1967; Dette and Linke, 1982; Holst-Wolf et al., 2016). The young participants who were 5–6 years of age in the current study may have had underdeveloped proprioceptive systems, which may have caused their less clearly defined categorization of bimodal presentations.

It is generally accepted that auditory discrimination is poorer and more variable in children than in adults (Buss et al., 2009; MacPherson and Akeroyd, 2014), and children’s lower psychometric scores are often related to poorer attention (Moore et al., 2008).

MSI requires sustained attention, and researchers have suggested that poor psychometric scores in children might be related to an attentional bias between the recruited senses in children vs. adults (Spence and McDonald, 2004; Alsius et al., 2005; Barutchu et al., 2009). For example, Barutchu et al. (2009) observed a decline in multisensory facilitation when auditory inputs were presented with a reduced signal-to-noise ratio. They suggested that the increased level of difficulty in performing the audiovisual detection task under high noise condition may be responsible for the degraded integrative processes.

If this attention bias might explain some of the between-group performance differences found when /e/-like somatosensory inputs were presented with /ø/-like auditory inputs (high level of difficulty), it would not justify differences between children and adults when the auditory and somatosensory channels agreed. The children showed decreased multisensory ability when both sensory inputs were compatible. Since difficulty level was reduced when multiple sensory sources were compatible, we should only have observed confusion in the children’s categorization when auditory and somatosensory information was incongruent. According to the intersensory redundancy hypothesis, MSI should be improved when information from multiple sources is redundant. Indeed, Bahrick and Lickliter (2000) suggested that concordance of multiple signals would guide attention and even help learning (Barutchu et al., 2010). In the current study, this multisensory facilitation was only found in the adult participants.

This latter observation and the fact that no significant differences in variability were found across experimental conditions make it difficult to link the dissimilar patterns of MSI found between the two groups to an attention bias in children. However, finding a greater variability in MSI in children in both conditions, combined with their distinct psychometric and categorical scores provides support for the concept that perceptual systems in school-aged children are not yet fully shaped, which prevents them from attaining adult-like categorization scores.

As speech processing is multisensory and 5- to 6-year-olds have already experienced it, it is not surprising that some differences, even typical MSI ones, were found between the two experimental conditions in children. Since even very young children recognize that various speech sensory feedback can be compatible—or not (Patterson and Werker, 2003; Yeung and Werker, 2013; Bruderer et al., 2015; Werker, 2018), the different behavioral patterns observed in this study suggest that some form of multimodal processing exists in school-aged children, but complete maturation of the sensory systems is needed to achieve adult-like MSI.

## Conclusion

When somatosensory input was added to auditory stimuli, it affected the categorization of stimuli at the edge of the categorical boundary for both children and adults. However, while the oral skin stretch manipulation had a defining effect on phonemic categories in adults, it seemed to have a blurring effect in children, particularly on the prototypical auditory stimuli. Overall, our results suggest that since adults have fully developed sensory channels and more experiences in MSI, they have stronger auditory and somatosensory integration than children.

Although longitudinal observations are not possible, two supplementary experiments in these participants has been conducted to further investigate how MSI takes place in speech processing in school-aged children and adults. These focus on the role of visual and auditory feedback.

## Data Availability Statement

The datasets generated for this study are available on request to the corresponding author.

## Ethics Statement

This study was carried out in accordance with the recommendations of “Comité institutionnel d’éthique de la recherche avec des êtres humaines (CIERH) de l’Université du Québec à Montréal [UQAM; Institutional review board of research ethics with humans of the Université of Québec in Montréeal (UQAM)]” with written informed consent from all subjects or their parent (for minor). All subjects or their parent gave written informed consent in accordance with the Declaration of Helsinki. The protocol was approved by the “Comité institutionnel d’éthique de la recherche avec des êtres humaines (CIERH) de l’Université du Québec à Montréal [UQAM; Institutional review board of research ethics with humans of the Université of Québec in Montréeal (UQAM)]”.

## Author Contributions

PT-F, TI and LM contributed to the conception and design of the study. PT-F collected data, organized the database and performed the statistical analysis (all under LM’s guidance). PT-F wrote the first draft of the manuscript. PT-F and LM were involved in subsequent drafts of the manuscript. PT-F, TI and LM contributed to manuscript revision, read and approved the submitted version.

## Conflict of Interest

The authors declare that the research was conducted in the absence of any commercial or financial relationships that could be construed as a potential conflict of interest.
